# A rare association of the absence of left pulmonary artery with ventricular septal defect, pulmonary hypertension, and more interestingly, patent ductus arteriosus in an adult patient: Case report and literature review

**DOI:** 10.1002/ccr3.9138

**Published:** 2024-07-09

**Authors:** Mozhgan Parsaee, Sedigheh Saedi, Soudabeh Behrooj, Elaheh Emami, Pegah Salehi, Ali Mohammadzadeh

**Affiliations:** ^1^ Echocardiography Research Center, Rajaie Cardiovascular Medical and Research Center Iran University of Medical Sciences Tehran Iran; ^2^ Rajaie Cardiovascular Medical and Research Center Iran University of Medical Sciences Tehran Iran

**Keywords:** patent ductus arteriosus, pulmonary hypertension, unilateral absence of pulmonary artery, ventricular septal defect

## Abstract

**Key Clinical Message:**

In this study, we introduced one of the rarest concomitants of the absence of left pulmonary artery (LPA), which was seen in our patient along with patent ductus arteriosus (PDA) and ventricular septal defect (VSD).

**Abstract:**

Unilateral absence of pulmonary artery (UAPA) is a congenital heart disease in association with other abnormalities such as tetralogy of Fallot and septal defects or isolated in 30% of cases and occurs in the right lung in two thirds of cases. Our case is a 33‐year‐old man who was hospitalized with symptoms of cough, shortness of breath, and hemoptysis. The echocardiography revealed a large ventricular septal defect, absent left pulmonary artery, and severe pulmonary hypertension (PH) along with patent ductus arteriosus. These findings were confirmed by CT angiography. This association has rarely been found in past studies. Due to PH and pulmonary infection, the patient was treated with intravenous prostaglandin and antibiotics. However, in cases of timely diagnosis and treatment of UAPA, fatal complications such as pulmonary hypertension, morbidity, and mortality are reduced. This case emphasizes the importance of awareness of this abnormality and its associated anomalies to enable early diagnosis and treatment.

## 1 INTRODUCTION

Unilateral absence of pulmonary artery (UAPA) is a condition that occurs when the sixth aortic arch fails to connect with the pulmonary trunk during the developmental process.^1^ UAPA is an uncommon congenital anomaly that can manifest independently or in conjunction with other congenital cardiac illnesses, such as tetralogy of Fallot (TF), coarctation of the aorta, and septal defects.^2^, ^3^ In contrast, over 30% of patients with UAPA do not have any related cardiovascular abnormalities.^4^ This condition is referred to as solitary UAPA. Two studies examining individuals with UAPA found that the median age of patients was 14.^2^, ^5^ The chest radiograph of patients with UAPA typically shows a small hemithorax, an ipsilateral absent hilar shadow, the absence of ipsilateral pulmonary artery, and ipsilateral hemi diaphragm elevation. To definitively diagnose UAPA, suspicious features on a chest radiograph can be confirmed with contrast‐enhanced computed tomography (CT) or magnetic resonance angiography (MRA).^6^ In this case, we presented an example of the association between absent left pulmonary artery (LPA), ventricular septal defect (VSD), and patent ductus arteriosus (PDA), which was rarely mentioned in previous studies. The treatment of UAPA does not have a specific guideline, and therapies such as revascularization of the PA to the peripheral branches in pediatric patients to angiographically embolization in massive hemoptysis and lobectomy or pneumectomy are recommended in non‐response patients.^7^

## 2 CASE HISTORY

The patient is a 33‐year‐old man with a history of a large VSD and PDA. He has been diagnosed with Eisenmenger syndrome since childhood and was treated for pulmonary hypertension (PH). The patient came to our center complaining of shortness of breath, cough, and hemoptysis. At the beginning of hospitalization, the vital signs were as follows: systolic blood pressure (SBP) = 98/49 mmHg, heart rate (HR) = 104 bpm, respiratory rate = 34, temperature = 37, lower limbs O_2_ saturation in room air = 66%, upper limbs O_2_ saturation in room air = 71% which reaches 81% with oxygen consumption.

In the physical examination, a continuous murmur was heard in the upper left sternal border and generalized rales in both lungs, clubbing of the fingers, and cyanosis was clear in the upper and lower limbs, which was especially higher in the lower limbs. Laboratory data were as follows: creatinine = 1 mg/dL, hemoglobin = 15.2 g/dL, platelet = 2,600,000/mm^3^, white blood cell (WBC) = 10,200 cell/mm^3^ with preferential percentage of neutrophil (85%) was seen, which was in favor of the presence of infection.

Sildenafil 50 every 12 h (BD), bosentan 125 BD, and furosemide 20 BD were evident in the patient's drug history.

## 3 METHODS

**FIGURE 1:**
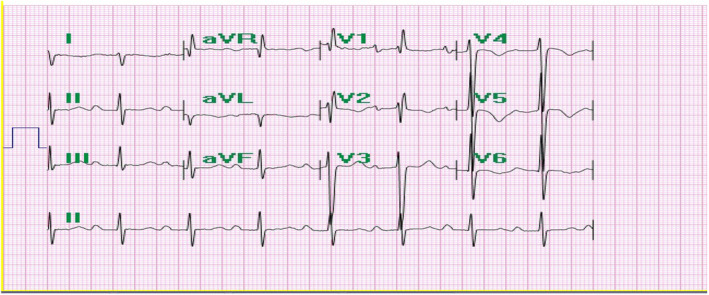
Sinus rhythm with right axis deviation and right ventricular hypertrophy in ECG.

In the ECG, there was evidence of sinus rhythm, right axis deviation, and right ventricular hypertrophy (RVH) (Figure 1).

**FIGURE 2:**
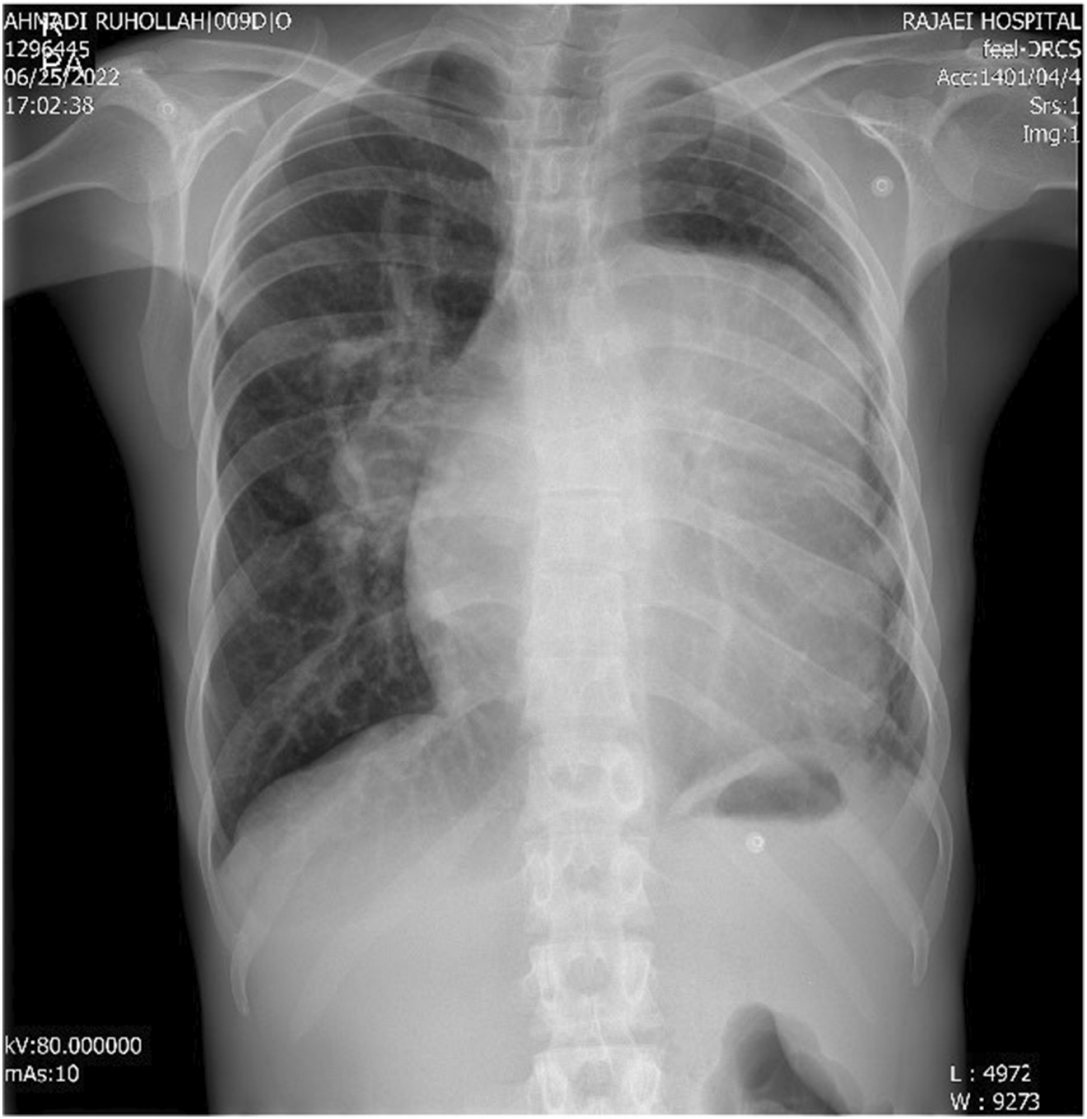
Chest X‐ray revealed cardiomegaly with prominent left ventricle and left atrium shadow, with prominent right pulmonary hilum and decreased left hemithorax volume.

Chest X‐ray (CXR) of the patient revealed that there was a prominent left atrium, left ventricle (LV), and ascending aorta and increased pulmonary vascular marking in the right hemithorax but very small left hemithorax (Figure 2).

Echocardiography was performed, and the results indicated the following findings:

1. Moderate enlargement of the left ventricle (LV) with a left ventricular ejection fraction (EF) of 35%.
2. Moderate right ventricular (RV) dysfunction and moderate right ventricular hypertrophy (RVH).
3. Severe pulmonary insufficiency.
4. Aneurysmal dilation of the main pulmonary artery (PA) with a diameter of 8 cm. The right pulmonary artery (RPA) measured 5.3 cm, and the left pulmonary artery (LPA) was absent.
5. A large patent ductus arteriosus (PDA) of 1.5 cm with a bidirectional shunt originating from the main pulmonary artery.
6. A large high muscular ventricular septal defect (VSD) of 2.2 cm with a bidirectional shunt.

**FIGURE 3:**
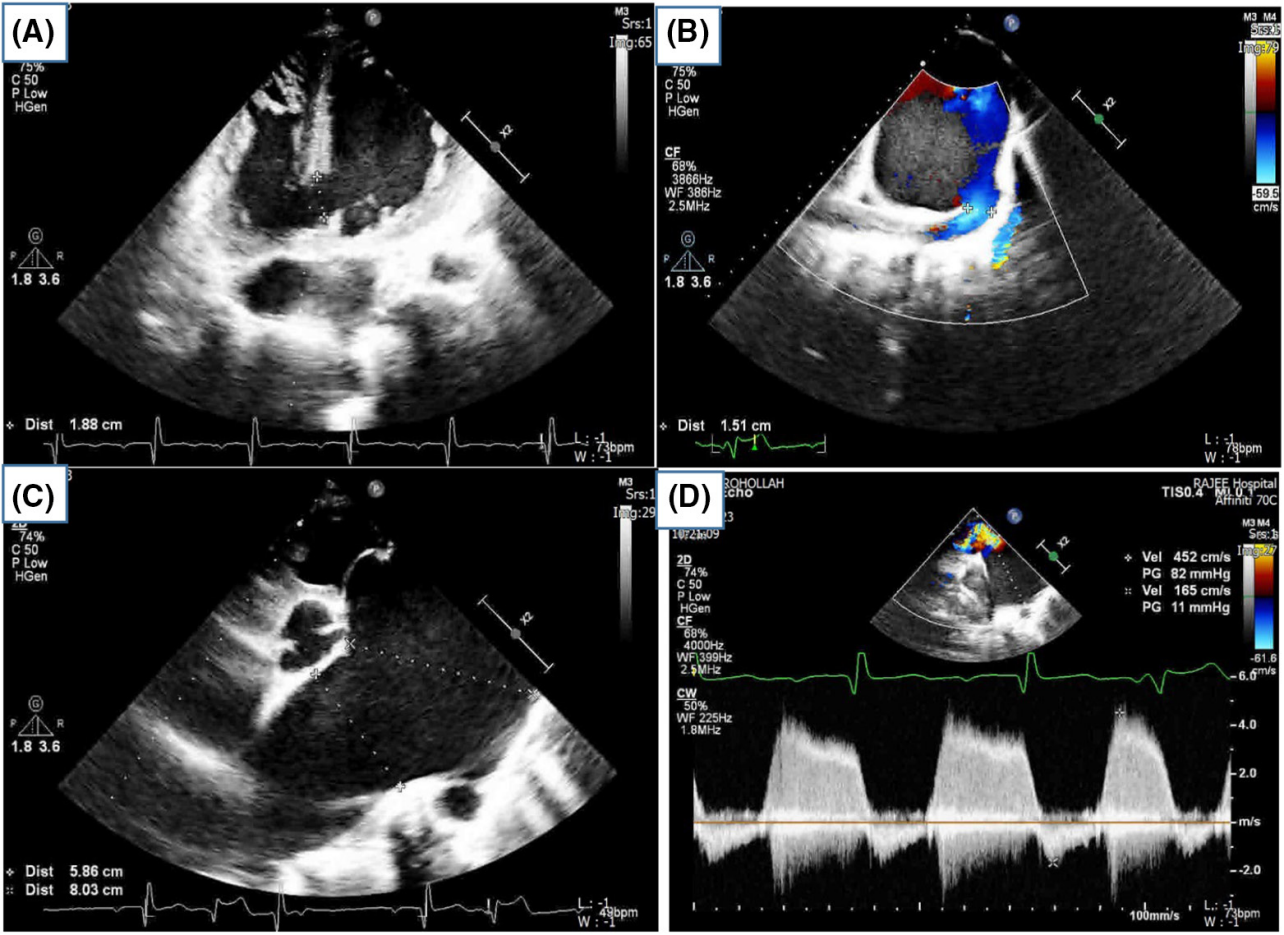
Patient's echocardiographic findings: (A) large high muscular ventricular septal defect (2.2 cm) with a bidirectional shunt in trans‐thoracic echocardiography (four chamber view); (B) large patent ductus arteriosus (1.5 cm) with a bidirectional shunt in trans‐thoracic echocardiography (short‐axis view); (C) aneurysmal main PA (8 cm), RPA (5.3 cm), and absent LPA in trans‐thoracic echocardiography (short‐axis view); (D) severe PH (mean PAP = 87 mmHg) in trans‐thoracic echocardiography (short‐axis view).

Patient's mean pulmonary artery pressure (mPAP) was 87 mmHg, which was classified as severe PH (Figure 3, Movies 1 and 2).

**MOVIE 1:** PDA bidirectional shunt in transthoracic echocardiography (short axis view).

**MOVIE 2:** Absent LPA in transthoracic echocardiography (short axis view).

**FIGURE 4:**
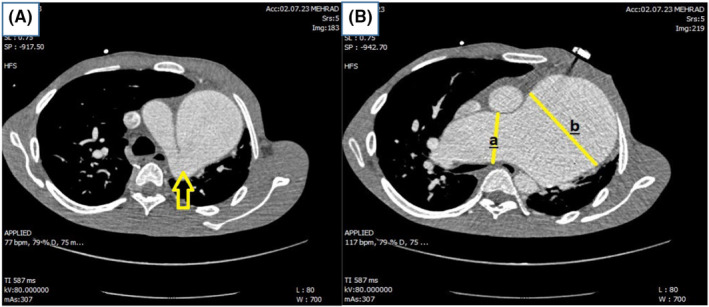
(A) PDA that was a connection between main PA and descending aorta (arrow) in this patient, (B) aneurysmal dilation of main PA (line b: 9 cm) and RPA (line a: 6.2 cm) in CT angiography.

Pulmonary computed tomography angiography (CTA) was performed, and the diagnosis of absent LPA was confirmed; the left lung was underdeveloped compared to the right lung and had mosaic attenuation. Left pulmonary infection that was confirmed in pulmonary CTA revealed ground glass opacities in left lung in favor of pulmonary infection (Figure 4).

## 4 CONCLUSION AND RESULTS

Finally, according to the diagnosis of severe PH and pulmonary infection, the patient underwent antibiotic treatment and conservative treatment with IV prostaglandin; so the symptoms improved and the patient was discharged with conservative anti‐pulmonary hypertension treatment, which continued with a higher dose than before. Although in the next follow‐up, unfortunately, the mPAP level did not decrease and no significant improvement was achieved, which is probably justified in terms of Eisenmmenger's physiology in this patient.

## 5 DISCUSSION

UAPA is a congenital disorder with a prevalence of 1 in 200,000 in adults, and has been reported along with other anomalies in past studies such as TF, atrial septal defect (ASD), VSD, translocation of great arteries, aortic coarctation and right aortic arch, truncus arteriosus, partial anomalous pulmonary venous, PDA, and its association with pulmonary atresia (PA) has also been reported and in some cases, it exists in isolation.^6^, ^7^ In the patient presented in this case, we found the association of left pulmonary atresia with PDA and VSD simultaneously, which was rare in previous articles.

From the embryonic point of view, UAPA is usually on the opposite side of the aortic arch, and due to the lack of growth of the proximal sixth aortic arch in the presence of an intact distal intrapulmonary branch, which is supplied with blood through the bronchial, intercostal, internal mammary, and subclavian arteries and sometimes even through the coronary arteries.^8^, ^9^

In the study of Ten Harkel and Ping Wang, in total, the symptoms of these patients were hemoptysis in 41%, exertional dyspnea in 41.5%, respiratory infection in 35.4%, bronchiectasis in 30%, lung tissue change and multiple bullas in 14%, exertional dyspnea is more common in people with PH than in people without PH, and in addition, 30% of UAPA patients have no symptoms or have a mild clinical course and remain asymptomatic until adulthood. The cause of PH in UAPA patients is the pushing of blood from absent PA to the rest of the PA, which, as a result of an increase in blood flow on the opposite side, causes shear stress on the endothelium and production of vasoconstrictive substances such as endothelin. In our patient, there were symptoms of shortness of breath during activity, self‐limiting hemoptysis that the patient's symptoms had worsened 3 years ago. The symptoms were previously attributed to PH and documented Eisenmenger's physiology since a young age, with a dominant right‐to‐left shunt through VSD and PDA, resulting in cyanosis, clubbing, and systemic desaturation. Eisenmenger syndrome is characterized by a triad of: (1) large intracardiac or extracardiac defects such as ASD, VSD, and PDA resulting in (2) systemic to pulmonary shunting and pulmonary arterial hypertension with shunt reversal, leading to right‐to‐left shunt or bidirectional shunting, causing (3) hypoxemia and cyanosis. All these conditions were present in our patient, leading to the diagnosis of Eisenmenger syndrome.^10^, ^11^ Hemoptysis in UAPA patients is due to the expansion of collateral vessels, which includes the venous system's unusually high pressure, but usually hemoptysis is self‐limiting^9^ The cause of recurrent infection in these patients is insufficient blood supply and chronic infection in the field of cilia dysfunction, which causes mucus to get stuck then eventually causes bronchiectasis in these patients.^12^

CXR findings in favor of UAPA include a small hemithorax on the affected side, an increase in the hemidiaphragm on the same side, and hyperinflation of the opposite lung.^6^, ^13^, ^14^ Also, small left hemithorax and right hemithorax hyperinflation were reported in our patient due to left UAPA.

The current diagnostic standard for these patients is angiography. However, due to advances in the field of CTA and MRA, a definite diagnosis can now be made using these two methods, as determined by the absence of RPA or LPA within 2 cm of the end area expected from the main PA. Angiography is only necessary in cases where embolization is needed for massive hemoptysis.^4^ After performing transthoracic echocardiography on the patient mentioned in this article and suspecting the diagnosis of absent LPA, he underwent CTA, and the diagnosis was confirmed.

The treatment of UAPA patients, according to the patient, is from revascularization in selected pediatric patients from the pulmonary branches on the side of affected PA to the pulmonary hilum to embolization in the case of hemoptysis, and finally lobectomy or pneumectomy in cases of infection or severe hemoptysis that does not respond to treatment.^4^, ^15^, ^16^ However, our case, which was a rare case of UAPA with PDA originating from main PA and VSD, was a candidate for medical treatment with vasodilators due to systemic PH, and no intervention was performed for him.

Early detection of UAPA and prompt and suitable treatments can enhance the prognosis and prevent complications before they occur, such as PH and reducing mortality and morbidity, so it is crucial that these patients are followed up regularly and their hemodynamics are observed with echocardiography.^7^

## AUTHOR CONTRIBUTIONS

**Mozhgan Parsaee:** Data curation; formal analysis; investigation; supervision; writing – review and editing. **Sedigheh Saedi:** Data curation; investigation; writing – review and editing. **Soudabeh Behrooj:** Formal analysis; investigation; writing – original draft. **Elaheh Emami:** Conceptualization; investigation; writing – original draft. **Pegah Salehi:** Conceptualization; data curation; investigation; methodology; supervision; writing – original draft; writing – review and editing. **Ali Mohammadzadeh:** Formal analysis; investigation; writing – review and editing.

## FUNDING INFORMATION

The authors received no financial support for the research, authorship, and/or publication of this article.

## CONFLICT OF INTEREST STATEMENT

The authors declared no conflicts of interest.

## CONSENT

Written informed consent was obtained from the patient to publish this report in accordance with the journal's patient consent policy.

## Data Availability

The data that support the findings of this study are available from the corresponding author (Pegah Salehi) on request.
